# Supplementary material for: Integrin-Driven Axon Regeneration in the Spinal Cord Activates a Distinctive CNS Regeneration Program
Source: J Neurosci. 2023 Jun 28;43(26):4775–94. doi: 10.1523/JNEUROSCI.2076-22.2023 (PMC10312060; doi:10.1523/JNEUROSCI.2076-22.2023)
Supplement: Extended Data Figure 7-1 — Genes upregulated in Cluster 1, magenta and black modules for the α9k1-crush (regeneration) group. These genes are associated with α9k1-driven axon regeneration. Expression changes in the clusters and modules are arranged by GO terms. The headings indicate the categories of GO terms, the italicised names in brackets are the names of individual GO terms. Further description is within the tables. The genes highlighted in green are found in the extended RAGs module from Chandran et al. (2016), demonstrating that there are very few RAGs in these modules/cluster. Download Figure 7-1, DOCX file. [file ns-JN-RM-2076-22-s01.docx]

**Extended Data Figure 7-1. Genes upregulated in Cluster 1, Magenta and Black modules for the α9k1-crush (regeneration) group**

These genes are associated with α9k1-driven axon regeneration. Expression changes in the clusters and modules are arranged by GO terms. The headings indicate the categories of GO terms, the italicised names in brackets are the names of individual GO terms. Further description is within the tables. The genes highlighted in green are found in the extended RAGs module from [Chandran et al., 2016](https://www.cell.com/neuron/fulltext/S0896-6273(16)00059-3?_returnURL=https%3A%2F%2Flinkinghub.elsevier.com%2Fretrieve%2Fpii%2FS0896627316000593%3Fshowall%3Dtrue), demonstrating that there are very few RAGs in these modules/cluster.

|  | **Cluster 1** | **Magenta Module** | **Black Module** |
| --- | --- | --- | --- |
| **Autophagy**  *(GO terms: process utilizing autophagic mechanism, and author-created group containing autophagy control molecules)* | Atg7, Becn1, Mfn2, Nbr1, Tbc1d14, Tollip, Vmp1, Vsp4b | Becn1, Ulk2, Nbr1, FIP200/Rb1cc1, Vmp1, Cttn, Mfn2, Tbc1d14, Tollip, Ubqln, Vps39, Usp10, Uvrag, Atp13a2, Zfyve1, Scoc, Tecpr1 | Pik3C3, OPTN, VCP, Paqr3, Atg7 |
| **ER/endosomes**  *(GO terms: endomembrane system organization and author-created ER group)* | Amfr, Atp11b, Ccdc47, Copa, Ddhd2, Dnajc10, Mfn2, Preb, Reep1, Sel1l, Trappc11, Zfyve27 | Mfn2, Reep1, Canx, Zfyve27, Lnpk, Vcp, Nacad, Amfr, Trappc11, Preb, Copa, Tmem30a, Ddhd2, Sel1l, Nploc4, Ccdc47 | n/a |
| **Transport/trafficking**  *(GO terms: establishment of localization, macromolecule localization, positive regulation of cellular component organization)* | Cdh4, Ddx3x, Eif4g2, Vps4b, Crk, Hif1a, Gnl3l, Itgb1, Actr3, Rgs2, Abi2, Skil, Mfn2, Add1, Rufy3, G3bp2, Anapc5, Cttn, Msn, Adam9, Becn1, Apbb1, Azin1, Prkci, Opa1, Kat7, Eif5a, Mief2, Psen1, Tppp, Dnm1l, Itga6, Syt3, Tmem30a, Rab3gap1, Cdc16, Ddhd2 | Raf1, Akt1, Ndel1, Zfyve27, Scamp1, Ywhaz, Fam21, Copa, Mfn2, Tbc1d14, Aktip, Pip5k1c, Trappc11, Reep1, Syt11, Pacsin2, Vps53, Elmo2, Ank2, Rab3gap1, Arf3 | Itga3, Ywhah, Trappc12, Ap2m1, Epb41l3, Cpeb1, Optn, Klhl20, Kif5c, Vcp, Rufy3, Copg1, Tbc1d9, Pdcd6ip, Ap4b1, Ap2b1, Tbc1d9b, Snx10, Tbc1d13, Nacad, Tbc1d10b, Rab9b, Rab11fip5, Cadps, Paqr3, Dennd1b, Ap3b2, Klc1, Exoc1 |
| **Cytoskeleton**  *(GO terms: cytoskeletal protein binding, cytoskeleton)* | Vps4b, Crk, Prkar1a, Rnf19a, Actr3, Abi2, Stk39, Dnaja1, Plk3, Mfn2, Add1, Hsph1, Cttn, Wdr13, Msn, Bcar1, Dis3l, Map4, Ank2, Dync1li2, Ssx2ip, Azin1, Prkci, Opa1, Fam110b, Glg1, Arhgef18, Prkacb, Psen1, Tppp, Dnm1l, Tubgcp2, Cdc16, Gnai1, Ddhd2 | Wdr1, Capza2, Pacsin2, Crk, Ndel1, Tppp, Dnm1l, Gsk3b, Mapk8ip3, Tubgcp2, Actr3, Ank2, Clasp2, Myo5a, Pip5k1c, Bicd2, Tbcd, Coro7, Camk2d, Clip2, Jakmip1, Capza1 | n/a |
| **Ubiquitin***  *(GO terms: protein ubiquitination, ubiquitin-protein transferase activity)*  **Ubiquitination key below* | Sel1l, Rnf19a, Rnf115, Anapc5, Amfr, Rnf125, Ube3c, Fbxl5, Spsb1, Rnf149, Rnf14, Chfr, Rnf145, Dcaf11, Lztr1, Pja2, Trim2, Rnf4, Cul1, Cbfb, Kctd9, Cdc16, Tt3 | *Amfr, Anapc5, Asb1, Cand1, Chfr, Cul1, Fbxo18, , Fbxl5, Klhl18, Mib2, Nedd4, Nub1, Pja1, Pja2, Rnf4, Rnf145, Rnf220, Spsb1, Tnfaip1, Trim2, Ube3b, Ube3c, Ubqln1, Usp22* | *Anapc2*, Anapc4, Asb13, Brap*,* Cul2, *Cul3,* Fzr1, Herc3, Keap1, Klhl20, Mkrn1, Rnf123, Trim36, Trpc4ap, *Uba1*, *UBC, Ube2e2* |

Ubiquitination Key

Yellow: SKF (SKP, Cullin, F- box) related molecules. E3 ligases, cullins, adaptors. Ubiquitylation at K48, K11

Grey: Ligases that ubiquitylate at K63, K11

Blue: Sumoylation transferases

Pink: E2 conjugating enzymes

: Adaptors, recognition molecules

No colour: Unknown type

*Italics:* mRNA present in sensory axons (Gumy et al., 2010)
