# Supplementary material for: Integrin-Driven Axon Regeneration in the Spinal Cord Activates a Distinctive CNS Regeneration Program
Source: J Neurosci. 2023 Jun 28;43(26):4775–94. doi: 10.1523/JNEUROSCI.2076-22.2023 (PMC10312060; doi:10.1523/JNEUROSCI.2076-22.2023)
Supplement: Extended Data Figure 7-2 — Genes upregulated in Clusters 2 and 3, pink and turquoise modules for the α9k1-naive group. The genes are associated with the expression of α9 integrin and kindlin-1 without dorsal root crush. Expression changes in the clusters and modules are arranged by GO terms. The headings indicate the categories of GO terms, the italicized names in brackets are the names of individual GO terms. Further description is within the tables. The genes highlighted in green are found in the extended RAGs module from Chandran et al. (2016), demonstrating that many of the genes upregulated by α9 integrin and kindlin-1 expression are previously identified RAGs. Download Figure 7-2, DOCX file. [file ns-JN-RM-2076-22-s02.docx]

**Extended Data Figure 7-2. Genes upregulated in Clusters 2 and 3, Pink and Turquoise modules for the α9k1-naïve group**

The genes are associated with the expression of α9 integrin and kindlin-1 without dorsal root crush. Expression changes in the clusters and modules are arranged by GO terms. The headings indicate the categories of GO terms, the italicised names in brackets are the names of individual GO terms. Further description is within the tables. The genes highlighted in green are found in the extended RAGs module from [Chandran et al., 2016](https://www.cell.com/neuron/fulltext/S0896-6273(16)00059-3?_returnURL=https%3A%2F%2Flinkinghub.elsevier.com%2Fretrieve%2Fpii%2FS0896627316000593%3Fshowall%3Dtrue), demonstrating that many of the genes upregulated by α9 integrin and kindlin-1 expression are previously identified RAGs.

|  | **Cluster 2** | **Cluster 3** | **Turquoise Module** | **Pink Module** |
| --- | --- | --- | --- | --- |
| **Immune response** *(GO terms: inflammatory response, immune system process, cytokine production)* | n/a | Ccl3, Csf1, Ccl4, Ccl11, Nfkbia, Il1rap, Ifngr2, Il6, Il1b, Vcam1, Usp18, Tnfrsf1a, Il4ra, Adcy7, Myd88, Cxcl10, Nfkbiz, Ccl2, Socs5, Socs3, Tnfaip6, Cebpb, Gbp5, Irf1, Ifih1, Eif2ak2, Irf7, Ripk2, Nfatc2, Ripk1 | Stat3, Bcr, Csf1, Sdc4, Fyn, Tnfaip3, Nos2, Nr1d1, Nfkbia, Ripk1, Golph3, Jak2, Il6, Casp1, Nfatc2, Tnfrsf1a, Il4ra, Myd88, Nfkbiz, Ccl7, Ccl2, Arid5a, Egr1, Thbs1, Pvr, Zfp36, Shb, Cebpb, Nfil3, Axl, Rab1a, Nos2, Hif1a, Ripk1, Jak2, Il6, Casp1, Il1b, Nfatc2, Tnfrsf1a, Il4ra, Fgfr1, Adcy7, Myd88, Ccl2, Arid5a, Egr1, Thbs1, Zfp36, Cebpb, Tnfaip8 | n/a |
| **Signaling**  *(GO terms: signalling receptor binding, Protein kinase binding, MAPK cascade, intracellular signal transduction)* | Adcyap1, Apoe, Atf4, Ednrb, Egfl8, Ghr, Gnai2, Grb10, Homer3, Nell1, Nr4a3, Nrtn, Ntrk2, Ptpn1, Rhob, Rhoc, Rit2, Serpine2, Src, Sumo1, Timp1, Traf4 | Araf, Cdk5rap3, Dusp1, Dusp2, Dusp5, Epha7, Fgfr1, Gadd45a, Gadd45b, Gadd45g, Map3k8, Mapk6, Mul1, Ptpn2, Rell1, Sh3rf1, Spry4, Tnip1, Trib1 | Axl, Stat3, Cd44, Tgfbr1, Bcr, Csf1, Gadd45b, Sdc4, Fyn, Tnfaip3, Sgk1, Rab1a, Nos2, Nr1d1, Nfkbia, Hif1a, Fos, Ripk1, Gadd45g, Golph3, Myc, Arc, Nr4a1, Rhoq, Syt4, Smad2, Jak2, Rabgef1, Ngef, Rgs1, Ralgds, Ptpra, Nfatc2, Crmp1, Ezh2, Tgfa, Tnfrsf1a, Smad1, Myd88, Prr5l, Nfkbiz, Sesn1, Akap2, Shb, Bdnf, Rasd1, Flrt3, Jun, Socs3, Mapk14, Agap1, Cebpb, Flot1, Rab7, Ripk1, Pim1 | Akt2, Mertk, Il1rap, Ptpn2, Rela, Pikfyve, Hdac4, Ddr2, Snap23, Ptpn1, Pld1, Nr4a3, Spp1, Pxn, Arhgef6, Farp2, Palm, Socs1, Mcl1, Dag1, Phlpp1, Plppr4, Rhob, Grik2, Dock1, Gpc6, Kalrn, Nck2, Mertk, Eps8, Sept4, Ptk2, Src, Socs1, Mcl1 |
| **Transcription factor**  *(GO terms: transcription regulatory region DNA binding, regulation of transcription by RNA polymerase II)* | Atf4 | Arid5a, Atf2, Atf3, Bach2, Bhlhe40, Btg2, Egr1, Fos, Fosb, Fosl1, Fosl2, Gabpb1, Irf1, Jun, Junb, Klf6, Mafk, Myc, Ncoa2, Nfe2l2, Nfil3, Nr1d1, Ppard, Smad1, Smad2, Srf, Stat3 | Klf6, Stat3, Mafk, Mdm2, Nr1d1, Nfkbia, Fos, Ripk1, Myc, Nr4a1, Fosl1, Il6, Irf1, Atf3, Atf2, Nfatc2, Fosl2, Bhlhe40, Smad1, ripk1, Arid5a, Egr2, Egr1, Zfp36, Junb, Cebpb, Nfil3 | Atf4 |
| **Regulation of localization and transport**  *(GO terms: early endosome, regulation of localization)* | n/a | Arc, Arrdc3, Ldlr, Rab1a, Rab21, Rabgef1, Sphk1, Stx12 | Mertk, Mical1, Rab10, Ptk2, Ddr2, Sdc3, Itgav, Snap23, Spp1, Dag1, Dmd, Rhob, Dock1, Kalrn | n/a |
| **Cell adhesion & cytoskeleton**  *(GO terms: integrin binding, cell adhesion, actin cytoskeleton, positive regulation of cell adhesion, biological adhesion)* | Cib1, F11r, Igf1, Itgav, Jam2, Mmp14, P4hb, Ptprz1, S1pr3, Spp1, Src, Bcan, Cadm1, Cd63, Cdh1, Cdh15, Cdh6, Cdhr4, Col12a1, Ctnna1, Ctnnd2, Dag1, Dlg1, Ephb1, Gpm6b, Itgav, Lsamp, Mdk, Ncam1, Ninj1, Notch1, Palld, Ptprt, Ttyh1 | Actn4, Capzb, Cttnbp2nl, Dlc1, Ermn, Kalrn, Klhl2, Llgl1, Llgl2, Mprip, Myo10, Nectin3, Rnd1, Sez6, Stau1, Swap70, Tpm3 | n/a | Ptk2, Itgav, Nr4a3, Spp1, Cited2, Dock1, Nck2, Cdh1, Mertk, Ptk2, Ptpn2, Sdc3, Lamc1, Itgav, Myo10, Nrcam2, Dlg1, Pxn, Nlgn3, Cdh11, Cdh8, Cdh6, Ncam1, Dag1, Dmd, Jam2, Ptprt, Rhob, Dock1, Nck2, Ptprz1, Pcdhgc3 |
| **Ubiquitin***  *(GO term: ubiquitin-protein transferase activity)*  **Ubiquitination key below* | n/a | n/a | Neurl1a, Peli1, Mdm2, Park2, Nedd4l, Arih1, Traf6, Itch, Rnf20, Rnf11, Trim21, Rnf128, Klhl2, Birc3, Rnf111, Fbxl2, Vhl, Siah2, Hace1, Rnf122, Rfwd2, Neurl3, Fbxo30, Rlim, Birc2, Ube2d3 | n/a |

Ubiquitination Key

Yellow: SKF (SKP, Cullin, F- box) related molecules. E3 ligases, cullins, adaptors. Ubiquitylation at K48, K11

Grey: Ligases that ubiquitylate at K63, K11

Blue: Sumoylation transferases

Pink: E2 conjugating enzymes
